# Minimally invasive approach: is this the future of aortic surgery?

**DOI:** 10.1007/s12055-021-01258-2

**Published:** 2021-12-06

**Authors:** Paolo Berretta, Michele Galeazzi, Mariano Cefarelli, Jacopo Alfonsi, Veronica De Angelis, Michele Danilo Pierri, Sacha M. L. Matteucci, Eugenio Alessandroni, Carlo Zingaro, Filippo Capestro, Alessandro D’Alfonso, Marco Di Eusanio

**Affiliations:** Cardiac Surgery Unit, Lancisi Cardiovascular Center, Polytechnic University of Marche, Via Conca 71, 60126 Ancona, Italy

**Keywords:** Thoracic aortic surgery, Minimally invasive cardiac surgery, Mini-Bentall operation, Mini-David operation

## Abstract

Median sternotomy incision has shown to be a safe and efficacious approach in patients who require thoracic aortic interventions and still represents the gold-standard access. Nevertheless, over the last decade, less invasive techniques have gained wider clinical application in cardiac surgery becoming the first-choice approach to treat heart valve diseases, in experienced centers. The popularization of less invasive techniques coupled with an increased patient demand for less invasive therapies has motivated aortic surgeons to apply minimally invasive approaches to more challenging procedures, such as aortic root replacement and arch repair. However, technical demands and the paucity of available clinical data have still limited the widespread adoption of minimally invasive thoracic aortic interventions. This review aimed to assess and comment on the surgical techniques and the current evidence on mini thoracic aortic surgery.

**Supplementary Information:**

The online version contains supplementary material available at 10.1007/s12055-021-01258-2.

## Introduction

Median sternotomy is considered the gold standard surgical access for thoracic aortic interventions; this approach provides optimal exposure of the entire proximal thoracic aorta—from the aortic root to the distal arch—and yields solid results. Over the last decade, however, minimally invasive cardiac surgery (MICS) has been increasingly accepted in the surgical community as a valid alternative to conventional full sternotomy, with benefits of decreased hospitalization, faster functional recovery, less blood transfusion and wound/sternal complications, and increased patients’ satisfaction. In particular, minimally invasive aortic valve replacement (AVR), through ministernotomy or anterior right mini-thoracotomy, has gradually been recognized as a less traumatic approach compared to median sternotomy, becoming the first-choice approach in several experienced centers [1]. The growing expertise in mini AVR techniques, coupled with increased patient demand for less invasive therapies, has motivated aortic surgeons to apply minimally invasive approaches to more challenging procedures, such as aortic root replacement and arch repair.

The present review aims to assess and comment on the surgical techniques and the current evidence on mini thoracic aortic surgery (MIAS).

## Surgical techniques

Preoperative screening and planning do not considerably differ from the conventional aortic procedures through median sternotomy. A careful review of computed tomography (CT) scan imaging is required to assess the anatomy of the aorta and the aortic vessels, and the aortic valve morphology to evaluate the degree and sites of either atheromatous or calcific aortic wall disease, in order to plan the most appropriate surgical incision, cannulation strategy, cross-clamp site, and organ protection method. Relative contraindications for MIAS are as follows: urgent and emergent surgery, previous cardiac surgery, severe chest wall deformities, active endocarditis, and concomitant procedures. In addition, MIAS should be approached cautiously in patients with giant aortic aneurysm.

Currently, two surgical accesses have been proposed for MIAS: the upper ministernotomy (MS) and the right mini-thoracotomy (MT).

### Upper MS

**Fig. 1:**
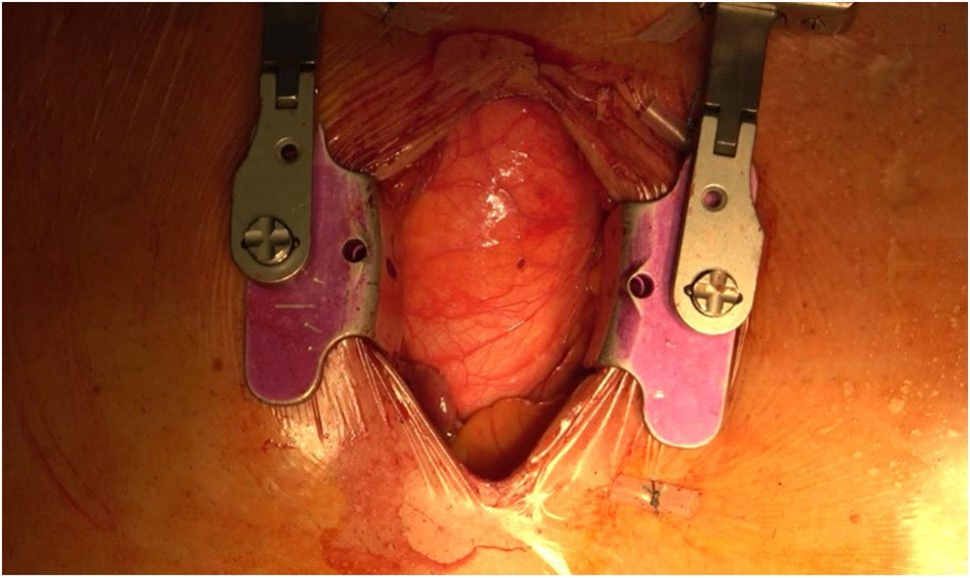
*J-*shaped upper mini-sternotomy

Upper MS is the most common less invasive access for thoracic aortic interventions. A 5–6-cm midline skin incision is made from the sternomanubrial junction to just above the 3^rd^ or the 4^th^ intercostal space (Fig. 1). Either a right-sided *J*-shaped or a left-sided *L*-shaped MS can be performed. The choice between the right or the left-sided MS is driven by the preprocedural CT-scan evaluation of the position of the aorta and by the type of planned intervention. As a rule, with the *J*-shaped MS, the access to the right superior pulmonary vein for the placement of the left ventricular vent is easier compared to the left-sided MS. Moreover, *J*-shaped MS avoids any potential injury to the left internal mammary artery, should it be used for coronary revascularization in the future. On the other hand, doing an *L*-shaped MS increases the exposure of the proximal and distal aortic arch as well as the aortic root. A *V* or inverted *T*-shaped upper MS has been also reported by some groups [2, 3]. While this access certainly improves visualization, it may reduce postoperative sternal stability and be associated with an increased risk of both left and right mammary arteries injury.

Conventional central cannulation can be routinely achieved for cardiopulmonary bypass (CPB) institutions in MS. Nevertheless, in patients with very large aneurysms, which virtually occupy the entire pericardial space and hamper safe and well controllable access to the central cannulation sites, or in patients who require hemiarch or arch replacement, an axillary or femoral approach should be preferred. Left ventricular venting is achieved through the right superior pulmonary vein or through the pulmonary artery.

**Fig. 2:**
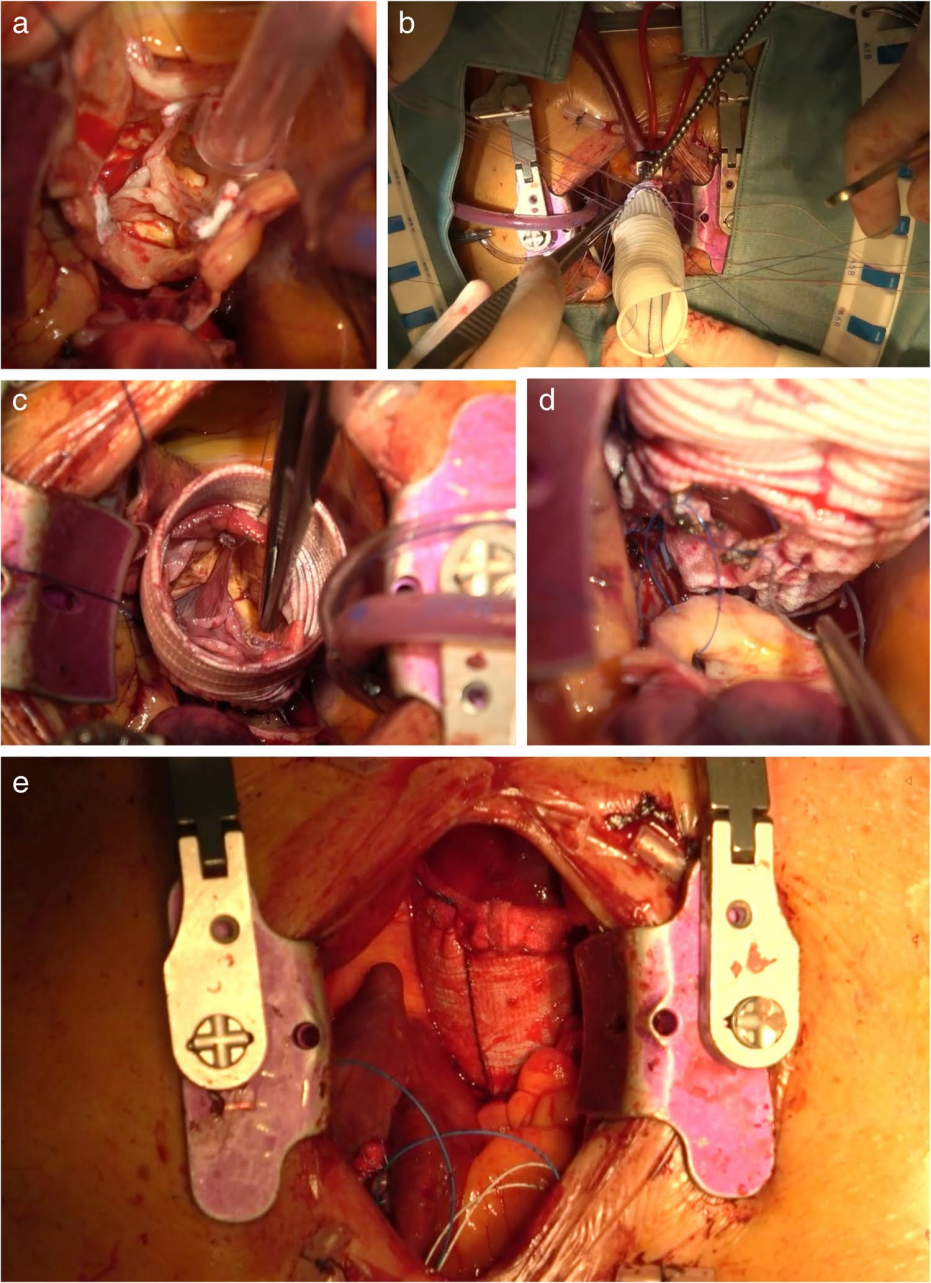
Mini-David operation through MS. **a** Aortic valve exposure. **b** Implantation of the Valsalva Dacron graft. **c** Aortic valve reimplantation. **d** Re-attachment of the left coronary ostium. **c** Final result

Using MS, aortic repair is performed in the same manner as a standard sternotomy access. Indeed visualization is similar to a full sternotomy, and no specialized techniques or equipment are required to perform the well-established operations of the ascending aorta and aortic root (Bentall [4], valve sparing [5], supracoronary ascending aorta replacement) (Fig. 2, Video 1) as well as of the aortic arch (hemiarch replacement [6] or total arch replacement with an elephant trunk or frozen elephant trunk construction [7, 8]). Nevertheless, it must be stressed that a meticulous surgical technique to secure impeccable hemostasis is essential in MIAS surgery. MS, however, allows for easy and safe conversion to full sternotomy in case of unexpected complications or unpredicted technical problems. In patients undergoing arch replacement, both antegrade selective cerebral perfusion (ASCP) or retrograde cerebral perfusion (RCP) methods can be used [9]. The advantages and drawbacks of such perfusion techniques were extensively reported in conventional aortic interventions and could be reasonably translated to MS approach [9]. Additionally, in patients undergoing mini arch surgery, RCP may provide improved exposure of the arch vessels, although there is constant flooding of venous blood return into the operative field, whereas ASCP yields a dry field with catheters potentially reducing visibility.

**Video 1** Mini-David operation through J mini-sternotomy approach

### MT

**Fig. 3:**
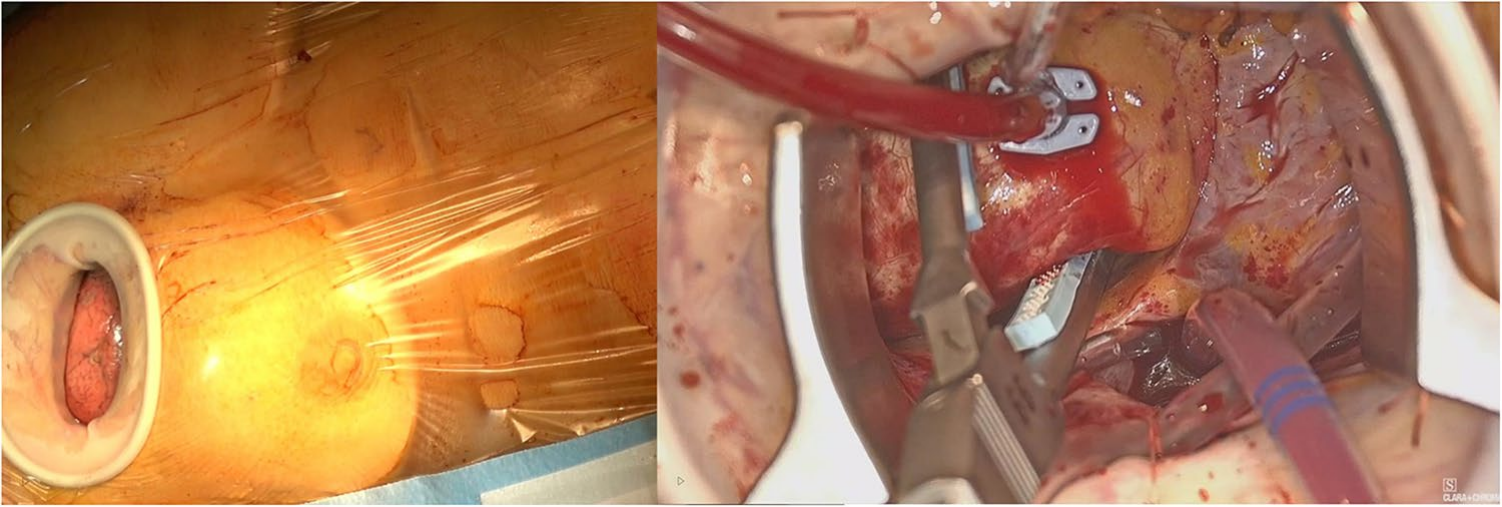
Anterior right mini-thoracotomy access

**Fig. 4:**
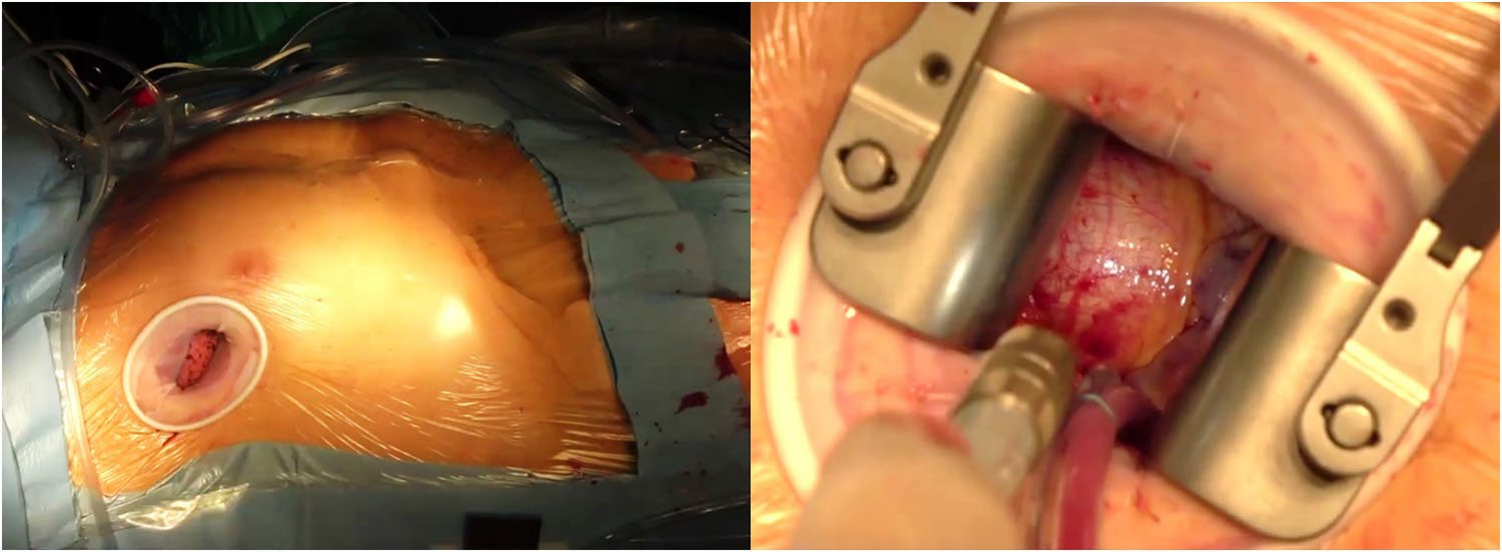
Lateral right mini-thoracotomy access

An alternative approach for MIAS is the right MT. While this approach is increasingly used for mini AVR interventions, only few groups described the feasibility of MT access in thoracic aortic surgery. Two MT accesses have been proposed for thoracic aortic interventions: the anterior right thoracotomy (ART) and the lateral/transaxillary MT [10, 11]. ART approach involves a 5–6-cm skin incision beginning 1 cm lateral to the sternum at the 2^nd^ or 3^rd^ intercostal space, transection of the internal mammary artery pedicle, and transection of the costochondral cartilage (Fig. 3). Using lateral MT, patients are positioned with the right arm elevated and the right lateral aspect of the chest elevated 30–45°; the 4^th^ (or the 3^rd^) intercostal space is entered via a skin incision made just lateral to the anterior axillary line extending 5–6 cm in length. No cartilage dislocation is needed (Fig. 4). This approach improves the exposition of the entire ascending aorta compared to ART, although the proximal arch and the aortic root can be further away. In single-lumen, endotracheal tube is used either in ART and lateral MT. CPB is established by means of femoral cannulation. Alternatively, the axillary artery can be cannulated for arterial inflow. The pericardium is opened over the aorta and extended down toward the inferior vena cava. Pericardial sutures are placed to provide adequate exposure and a left ventricular vent is placed through the right superior pulmonary vein. If the intervention is limited to replacing only the ascending aorta, the native aorta is cross clamped distally, and the proximal anastomosis is performed first in the usual fashion, followed by distal anastomosis. This sequence seems to facilitate visualization in the MT approach. Aortic cross-clamping is achieved using a minimally invasive clamp with a retractable shaft placed directly through the incision or using a Chitwood Debakey aortic clamp positioned through a separate 5-mm incision in the third interspace on the anterior-mid axillary line. In patients who require root replacement, after resection of the ascending aorta, the aortic root is exposed with 3 stay sutures, the coronary buttons are prepared, and the remaining portion of the aorta and sinus are resected. The intervention is then completed as usual. In MT interventions, long-shafted minimally invasive instruments as well as knot setter or automated fastener device (Cor Knot System, LSI Solutions, NY, USA) are needed to facilitate suturing and valve implantation. Additionally, the use of a camera through a port placed lateral to the incision has been proposed to enhances visualization of the aortic root in patients receiving ART [11].

In patients undergoing hemiarch replacement, cerebral protection can be exclusively performed via deep hypothermic circulatory arrest and RCP [9]. The latter is obtained by means of a venous cannula placed into the superior vena cava through a port site positioned several interspaces below the MT. In hemiarch interventions, after removal of the distal aorta, additional pericardial sutures are placed at the aortopericardial insertion to provide the necessary exposure of the aortic arch. These sutures are mandatory to obtain adequate exposure of the arch [10]. No case of total arch replacement using MT accesses has been reported, thus far.

## Current evidence

MIAS was introduced in the early 2000s as an alternative to conventional sternotomy, but with a potential benefit of reduced trauma and quicker recovery [12, 13]. Since then, a number of single-center retrospective studies have reported the feasibility of performing mini aortic interventions (Tables 1, 2, and 3).

**Table 1:** Summary of study characteristics

Author	Year	Study period	Study design	No. of patients	Age (years) Mean ± SD/ median (IQR)	Operative risk Mean ± SD/ median (IQR)	Aortic pathology %	Urgency/emergency %
**Root**/**ascending aorta surgery**								
Elghannam et al. [28]	2020	2011–2017	Retrospective	47	57.9 ± 10.5	ES II: 3.5 ± 2.5	Aneurysm: 96 Chronic aortic dissection: 4	-
Staromlynski et al. [3]	2020	2011–2018	Retrospective	167	64.1 ± 11.3	ES II: 2.58 ± 3.26	NA	-
Hillebrand et al. [29]	2018	2012–2016	Retrospective, comparative study	33	55.68 ± 13.24	ES II: 6.39 ± 3.57 Log ES I: 10.79 ± 13.4	Aneurysm: 100	-
Shrestha et al. [27]	2015	2011–2014	Retrospective	26	47.6 ± 13	NA	Aneurysm: 100	-
Sun et al. [13]	2000	1999	Retrospective study	8		NA	Aneurysm: 100	-
Monsefi et al. [14]	2018	1991–2016	Retrospective, propensity matched	120	56 ± 14	NA	Aneurysm: 100	-
Abjigitova et al. [30]	2018	1998–2016	Retrospective	26	60 (50–63)	NA	Aneurysm: 88.5 Endocarditis: 7.7	19.2
Mikus et al. [20]	2017	2010– 2015	Retrospective	53	63 (51–73)	Log ES I: 9.06 ± 5.8	Aneurysm: 96.2 Endocarditis 3.8	3.8
Wachter et al. [15]	2016	2007–2012	Retrospective, propensity matched	117	56.5 ± 13.6	ES II: 3.1 (1.8–3.4)	Aneurysm: 100	-
Deschka et al. [31]	2013	2007–2010	Retrospective	50	62.5 ± 8.9	Log ES I: 10.0 ± 6.5	Aneurysm: 100	-
Tabata et al. [16]	2007	1996–2005	Retrospective, propensity matched	128	53.8 ± 13.9	NA	Aneurysm: 65 Chronic aortic dissection: 7.8 Endocarditis: 4.7	3.9
Perrotta et al. [32]	2008	1997–2005	Retrospective	40	53 ± 14	NA	Aneurysm: 92.5 Acute aortic dissection: 7.5	7.5
Svensson et al. [33]	2001	NA	Retrospective	54	60.8 ± 16.9	NA	Aneurysm: 70.4 Aortic dissection: 29.6	NA
Levack et al. [17]	2016	1995–2014	Retrospective, propensity matched	568	55 ± 14	NA	Aneurysm: 100	-
Hastaoglu et al. [34]	2018	2010–2015	Retrospective, propensity matched	54	57.9 ± 12.5	NA	NA	-
Johnson et al. [35]	2018	2016–2018	Retrospective	7	62	NA	NA	-
Lamelas et al. [21]	2018	2009–2014	Retrospective, propensity matched	74	62.7 ± 13.06	NA	NA	-
**Arch surgery**								
Iba Y et al. [22]	2019	2016–2018	Retrospective	22	76 (72–82)	ES II: 3.12 (2.06–6.93)	Aneurysm: 91 Chronic aortic dissection: 9	-
Risteski et al. [7]	2020	2007–2019	Retrospective	123	66 ± 12	NA	Aneurysm: 74.8 Chronic aortic dissection: 17.1 Pseudoaneurysm: 4.9 Porcelain aorta: 3.3	-
Goebel et al. [23]	2017	2008–2015	Retrospective	21	69.3 ± 14.4	Log ES I: 17.0 ± 7.2	Aneurysm: 86 Chronic aortic dissection: 14	-

*SD* standard deviation, *IQR* interquartile range, *ES* European system for cardiac operative risk evaluation (EuroSCORE), *NA* not available

**Table 2:** Summary of operative characteristics

Author	Surgical access	Type of intervention %	Associated procedures %	CPB time (min) Mean ± SD/ median (IQR)	Myocardial ischemia time (min) Mean ± SD/ median (IQR)	Circulatory arrest % (time) Mean ± SD/ median (IQR)	Cerebral protection method	Conversion to full sternotomy %
**Root/ascending aorta surgery**								
Elghannam et al. [28]	J ministernotomy (to 3rd or 4th ICS)	Bentall: 77 Valve sparing: 23	-	174 ± 54.8	133 ± 33.1	-	-	-
Staromlynski et al. [3]	V-shaped ministernotomy (to 3rd or 4th ICS)	Isolated ascending aorta: 75 Bentall: 15 Valve sparing: 9.6	AVR: 26 Aortic valve repair: 4.2 Hemiarch/proximal arch replacement: 0.6	152.0 ± 46.8	101.8 ± 36.8	0.6%	NA	0.6
Hillebrand et al. [29]	J ministernotomy (to 4th ICS)	Bentall: 100	Mitral valve surgery: 9 Tricuspid valve surgery: 6 PFO closure: 3	166.12 ± 40.61	122.24 ± 27.42	-	-	-
Shrestha et al. [27]	J ministernotomy (to 3rd ICS)	Valve sparing: 100	Aortic valve repair: 42.3 Hemiarch/proximal arch replacement: 15.4 CABG: 3.9	175.8 ± 41.9	163 ± 24.5	15.4% (22.5 ± 13.6 min)	ASCP	-
Sun et al. [13]	*J*- or *L*-shaped ministernotomy (to 4th ICS)	Valve sparing: 100	-	78.1 ± 6.9	58.2 ± 5.2	-	-	NA
Monsefi et al. [14]	J ministernotomy (to 4th ICS)	Valve sparing: 100	Hemiarch/proximal arch replacement: 13 Total arch replacement: 18 Mitral valve surgery: 10 Tricuspid valve surgery: 2 CABG: 4 ASD closure: 2	183 ± 46	136 ± 31	31%	ASCP	-
Abjigitova et al. [30]	J or inverted-T ministernotomy	Bentall: 100	-	169 (156–188.5)	148 (131.3–160.3)	-	-	-
Mikus et al. [20]	J ministernotomy (to 3rd ICS)	Bentall: 100	-	84 (75–103)	73 (64–89)	-	-	NA
Wachter et al. [15]	J ministernotomy (to 3rd or 4th ICS)	Valve sparing: 100	Aortic arch replacement: 1.7 AF ablation: 4.3 Septal myectomy: 0.9	164.9 ± 32.9	131.3 ± 21.9	1.7%	NA	-
Deschka et al. [31]	L-shaped ministernotomy	Isolated ascending aorta: 100	AVR: 30 Hemiarch/proximal arch replacement: 22 Total arch replacement: 6	140.9 ± 35.3	95.5 ± 27.5	28% (24.3 ± 9.6 min)	DHCA, ASCP	-
Tabata et al. [16]	J ministernotomy (to 3rd or 4th ICS)	Isolated ascending aorta: 46.9 Root: 52.3	Hemiarch/proximal arch replacement: 5.5	152 ± 57	108 ± 42	NA (38 ± 25 min)	DHCA, ASCP	-
Perrotta et al. [32]	J or inverted-T ministernotomy (to 3rd or 4th ICS)	Bentall: 100	Hemiarch/proximal arch replacement: 5 CABG: 2.5	154 ± 41	107 ± 20	7.5%	DHCA, ASCP	-
Svensson et al. [33]	J ministernotomy (to 3rd or 4th ICS)	Isolated ascending aorta: 72 Bentall: 28	AVR: 48 Arch replacement: 33.3 ASD repair: 1.8 Mitral valve surgery: 1.8 Thoracoabdominal aorta repair: 1.8	132 ± 59	91 ± 45	35.1% (20 ± 17 min)	ASCP, RCP	-
Levack et al. [17]	J ministernotomy (to 4th ICS)	Isolated ascending aorta: 80.8 Bentall: 13 Valve sparing: 6.3	AVR: 47.9 Aortic valve repair: 24.4	70 ± 26	55 ± 21	-	-	1.9
Hastaoglu et al. [34]	J ministernotomy (to 4th ICS)	Isolated ascending aorta: 80 Bentall: 20	AVR: 40	97.09 ± 23.32	75.69 ± 22.75	11% (15.80 ± 5.72 min)	ASCP	-
Johnson et al. [35]	ART	Bentall: 100	-	202.9 ± 47.8	161.9 ± 32.4	100% (26.6 ± 11 min)	DHCA	NA
Lamelas et al. [21]	ART or lateral right thoracotomy	Isolated ascending aorta: 100	-	183 (153–205)	141 (113–164)	NA 37 (33–43) min	RCP	6.8
**Arch surgery**								
Iba Y et al. [22]	L or reversed-T ministernotomy (to 3rd or 4th ICS)	Hemiarch/proximal arch replacement: 9 Total arch replacement: 86.4 FET: 4.5	AVR: 5 Aortic valve repair: 5	214 (183–228)	109 (94–125)	100% 50 (38–56) min	ASCP	5
Risteski et al. [7]	L-shaped ministernotomy (to 4th ICS)	Hemiarch/proximal arch replacement: 55.3 Total arch replacement: 9.7 FET: 35	Aortic valve surgery: 33.3 Bentall: 7.3 Valve sparing: 25.2	178 ± 44	116 ± 36	100% (39 ± 12 min)	ASCP	-
Goebel et al. [23]	J or L-shaped ministernotomy (to 3rd or 4th ICS)	Hemiarch/proximal arch replacement: 85.8 FET: 14.3	AVR: 14.3 Bentall: 9.5 Valve sparing: 47.6	168.7 ± 41.7	115.6 ± 39.0	100% (26.4 ± 23.6 min)	ASCP	4.7

*SD* standard deviation, *IQR* interquartile range, *ICS* intercostal space, *AVR* aortic valve replacement, *AF* atrial fibrillation, *CPB* cardiopulmonary bypass, *ART* right lateral thoracotomy, *FET* frozen elephant trunk, *CABG* coronary artery bypass graft, *ASD* atrial septal defect, *PFO* persisting foramen ovale, *ASCP* anterograde selective cerebral perfusion, *RCP* retrograde cerebral perfusion, *DHCA* deep hypothermic circulatory arrest

**Table 3:** Summary of early and mid-term outcomes

Author	Mortality %	Stroke %	Bleeding %	Ventilation time (min) Mean ± SD/ median (IQR)	AKI %	New onset AF %	Wound complications %	ICU stay (days) Mean ± SD/ median (IQR)	In-hospital stay (days) Mean ± SD/ median (IQR)	Mean FU (months) Mean ± SD	Survival %	Freedom from redo %
**Root/ascending aorta surgery**												
Elghannam et al. [28]	In hospital: - 30-day: -	NA	4.2	10 (7.5–13.5)	NA	NA	Superficial: 2 Deep: –	1.9 ± 1.3	11.8 ± 4.4	NA	2y: 93.7	2y: 93.8
Staromlynski et al. [3]	In hospital: 1 30-day: 1	0.6	7.2	NA	4.8	NA	Deep: 1.3	2 (1–3)	NA	3.1	3y: 95	2y: 99
Hillebrand et al. [29]	In hospital: NA 30-day: 3	3	6	25.85 ± 65.52	NA	NA	Deep: –	2.45 ± 3.43	13.36 ± 9.27	NA	NA	NA
Shrestha et al. [27]	In hospital: - 30-day: -	–	3.2	12 ± 7.2	–	NA	NA	1.3 ± 0.6	10.4 ± 6.8	15.9 ± 10.7	NA	NA
Sun et al. [13]	In hospital: - 30-day: -	NA	12.5	14.8 ± 5.6		NA	–	3.0 ± 0.5	12.1 ± 5.4	3	100	NA
Monsefi et al. [14]	In hospital: - 30-day: NA	1	8	NA	NA	NA	Superficial: 1	1.1 ± 0.7	NA	36 ± 24	100	NA
Abjigitova et al. [30]	In hospital: - 30-day: -	–	–	NA	–	19.2	NA	3 (2–4.75)	6.5 (5–11)	NA	NA	NA
Mikus et al. [20]	In hospital: - 30-day: -	NA	6	13.2 ± 19	2	17	NA	1.9 (1.7–3.6)	8 (7–12.75)	NA	NA	NA
Wachter et al. [15]	In hospital: - 30-day: -	NA	9.4	10.2 ± 21.8	13.7	12	Deep: 0.9	1.9 ± 3.6	10.4 ± 5.5	31 ± 18	5y: 99	5y: 88
Deschka et al. [31]	In hospital: - 30-day: NA	2	2	28.6 ± 30.9	2	32	Deep: 2	2.1 ± 2.1	11 ± 6.5	NA	NA	NA
Tabata et al. [16]	In hospital: - 30-day: -	0.8	1.6	NA	–	NA	Deep: 0.8	NA	5 (range 3–21)	NA	5y: 97.2	NA
Perrotta et al. [32]	In hospital: 2.5 30-day: 2.5	5	2.5	52.7 ± 185.1	NA	22.5	NA	3.3 ± 8.2	9.3 ± 7.2	38.4 ± 31	5y: 90.6	NA
Svensson et al. [33]	In hospital: 3.7 30-day: NA	3.7	7.4	NA	1.9	NA	NA	1.8 ± 1.9	6.7 ± 3.7	NA	NA	NA
Levack et al. [17]	In hospital: 0.18 30-day: NA	0.7	3.2	NA	0.7	NA	Deep: –	1 (0.8–2)	5.2 (4.1–7.2)	NA	NA	NA
Hastaoglu et al. [34]	In hospital: - 30-day: NA	NA	4.4	3.67 ± 0.8	NA	NA	NA	NA	4.93 ± 0.91	NA	NA	NA
Johnson et al. [35]	In hospital: - 30-day: -	–	14	10.6 ± 6.9	–	14	Superficial: 14	1.33 ± 0.77	4.1 ± 0.9	NA	NA	NA
Lamelas et al. [21]	In hospital: NA 30-day: 2.7	–	–	9.3 (3.95–17.95)	1.4	20.3	–	1.27 (0.94–2.71)	5 (4–7)	NA	NA	NA
**Arch surgery**												
Iba Y et al. [22]	In hospital: - 30-day: -	–	3	–	–	32	–	3 (3–6)	23 (17–39)	NA	NA	NA
Risteski et al. [7]	In hospital: NA 30-day: 3.3	4.9	4	8.5 ± 3.3	9.8	NA	–	1.6 ± 0.6	7 ± 2	33.2 ± 16.2	5y: 80	NA
Goebel et al. [23]	In hospital: 1 30-day: NA	–	4.8	9 (6–46.5)	4.8	NA	–	1 (1–4.5)	NA	NA	NA	NA

*SD* standard deviation, *IQR* interquartile range, *NA* not available, *AKI* acute kidney injury, *AF* atrial fibrillation, *ICU* intensive care unit, *FU* follow-up

### Ascending aorta and root surgery

Current evidence on MIAS is limited to retrospective observational studies since no well-powered randomized controlled trial comparing less invasive approaches and conventional surgery has been performed (Table 1). The largest single-center series on mini ascending and root interventions showed that MIAS is a safe and efficacious procedure and is associated with excellent outcomes, in selected patients [3, 17, 20, 22, 25]. Early mortality ranges from 0 to 1%, with a stroke rate of 0.6–1%. Although only few series reported on the long-term outcomes, the available data seem to confirm the satisfactory results observed in the short term, with a 5-year survival rate ranging from 97 to 99% [15, 16].

Presently, there is still a paucity of comparative data or propensity-score matched analyses on MIAS. When comparing mini ascending aorta and root interventions to conventional surgery, observational studies revealed similar clinical outcomes in terms of mortality and major postoperative complications [18, 19]. Nevertheless, less invasive approaches were associated with less blood transfusions, shorter intensive care unit (ICU) and hospital stays, lower costs, and better cosmesis [14–17, 20, 21].

One of the main concerns regarding less-invasive operations is the perception that the increased technical demands of working through a confined space may translate into prolonged operative times. This assumption seems not to be confirmed by the available data [18]. The largest propensity-matched analysis from the Cleveland group including 483 pairs [17] showed that patients receiving J-MS were associated with shorter average cross-clamp time (55 vs. 70 min.) and CPB time (70 vs. 87 min) compared with those receiving conventional surgery (*p* < 0.001). Furthermore, while no difference was found in the incidence of in-hospital mortality and postoperative stroke, patients undergoing MIAS had shorter median ICU (24 vs. 26 h) and hospital length of stay (5.2 vs. 6 days) (*p* < 0.001). These results translated into 6% less operative and postoperative direct technical costs in the minimally invasive group.

The current evidence base on MIAS was recently assessed in a metanalysis comparing 1101 patients who underwent less invasive interventions vs. 1405 patients who underwent conventional sternotomy approach [18]. While no differences in major clinical outcomes between the 2 study cohorts were observed, the meta-analysis confirmed shorter CPB and cross-clamp times and reduced lengths of stay for patients undergoing MIAS. Moreover, MIAS was associated with reduced bleeding and renal impairment compared with conventional surgery. It has to be noted, yet, that the overall quality of evidence was very low.

### Arch surgery

Reports on minimally invasive arch surgery are scarce including only occasional small case series and no comparative study (Table 1) [7, 22, 23]. The published studies seem to support the feasibility and the efficacy of mini arch interventions. In the largest series on mini arch surgery, Risteski et al.[7] reported no conversion to full sternotomy in 123 consecutive patients undergoing aortic arch repair through MS access. Early mortality was 3.3% with an incidence of permanent and temporary neurologic deficit of 4.9% and 8.1%, respectively. A frozen elephant trunk repair was performed in 43 (33%) patients with a spinal cord injury rate of 3.3%. At 5 years, survival was 80% and freedom from reoperation was 96%. These results confirmed that minimally aortic arch repair through an upper MS can be performed safely, with outcomes well comparing to those reported in conventional aortic arch series [24–26]. Undoubtedly, further larger clinical trials are needed to collect more robust evidence in the setting of mini aortic arch procedures.

## Ancona’s experience

Between September 2016 and March 2021, 102 patients underwent minimally invasive root or ascending thoracic aorta repair at Lancisi Cardiovascular Center in Ancona, Italy. The median age was 70 years (interquartile range (IQR) 58–77), with a male predominance of 68.6% (*n* = 70). The median EuroSCORE II was 1.88% (IQR: 1.2–3.1). The indication for surgery was a degenerative aneurysm in all patients. Surgical procedures included isolated ascending aorta replacement (*n* = 32, 31.4%), AVR + ascending aorta replacement (*n* = 42, 41.2%) and aortic root replacement (*n* = 28, 27.5%) using Bentall (*n* = 19) or David (*n* = 9) technique. All procedures were performed through a *J*-or *L*-shaped MS and no patient required conversion to full sternotomy. Early results were excellent with no in-hospital death or stroke. Main complications included advanced acute kidney injury (stage 2–3) (2.9%), respiratory insufficiency (2%), postoperative myocardial infarction (2%), and bleeding (1%). The median ventilation time was 6 h (IQR: 4–10) with 12% of patients extubated directly in the operating room. ICU and hospital length of stay were 24.5 h and 6 days, respectively. At 1 year, the overall survival rate was 99% ± 0.1%.

## Future perspective

Surgery of the thoracic aorta has traditionally been performed via a standard full sternotomy incision. Nevertheless, with the popularization of MICS techniques, new observations regarding the treatment of patients with the thoracic aortic disease have arisen. MICS is a burgeoning field; as our experience with less invasive techniques is growing, more technically complex and demanding patients can be treated through less invasive approaches. With MICS and catheter-based techniques becoming the first-choice approach for valve interventions, patients increasingly request less invasive operations also in the setting of thoracic aortic pathologies. Currently, however, a considerable number of surgeons are still skeptical about MIAS, claiming that smaller incisions may lead to poor exposure and worse outcomes in such complex operations. New techniques have to provide the same safety and efficacy as the conventional approach. The preliminary data on MIAS we have assessed in this review indicated that MIAS can be performed with results, at very least, comparable to those of conventional surgery, in selected patients. Nevertheless, it must be pointed out that the majority of such results come from high-volume experienced aortic centers and cannot be generalized to the whole cardiac surgery community, at present. As with any new procedure, a learning curve for MIAS exists and may influence outcomes. The presence of surgeons experienced in thoracic aortic surgery and well trained to perform minimally invasive valve operations coupled with a “step-by-step” method of progressing gradually from simpler operations (isolated ascending aorta replacement) to technically more complex aortic root or arch surgery is mandatory to perform MIAS safely and expeditiously as conventional surgery [27].

Available data indicate that patients receiving MIAS are associated with shorter ICU and hospital length of stay [18]. Shortening patient recovery has become a key element to evaluate the results of contemporary surgery; rapid recovery provides greater patient satisfaction and reduces hospital costs [17]. Thus, the question arises: if the *same quality* of operation can be performed through a less traumatic approach, resulting in shorter hospital stays and lower overall costs, why should it not become the standard of care? We believe we currently stand on the brink of an innovative time when less invasive surgical techniques are changing the way we approach thoracic aortic disease. Although stronger clinical data are still needed, the preliminary data on MIAS are definitely encouraging. Nevertheless, caution must be stressed because conventional aortic interventions have proven excellent early and long-term outcomes and must remain our measure for comparison.

## Conclusions

Minimally invasive thoracic aortic interventions can be performed successfully without compromising the proven efficacy and safety of the conventional approach. The current evidence shows encouraging results in selected patients operated on in selected institutions. Nevertheless, technical demands and a paucity of comparative data are still limiting the widespread adoption of MIAS. Thus, further clinical trials are required to validate the use of MIAS techniques on a large scale.

## Supplementary Information

ESM 1 (mp4 134 mb)

## Data Availability

Not applicable.
